# Methyl *N*-[(4-chloro­phen­yl)(3-methyl-5-oxo-1-phenyl-4,5-dihydro-1*H*-pyrazol-4-yl­idene)meth­yl]glycinate

**DOI:** 10.1107/S1600536809027858

**Published:** 2009-07-22

**Authors:** Xin Zhang, Meng Huang, Cong Du, Dan Chen

**Affiliations:** a College of Chemistry and Life Science, Tianjin Normal University, Tianjin 300387, People’s Republic of China

## Abstract

The title compound, C_20_H_18_ClN_3_O_3_, is in an enamine–keto form, stabilized by two strong intra­molecular N—H⋯O hydrogen bonds. The pyrazole ring is oriented at dihedral angles of 4.13 (3) and 85.60 (3)° with respect to the aromatic rings. The dihedral angle between the aromatic rings is 81.79 (3)°. In the crystal structure, inter­molecular C—H⋯O hydrogen bonds link the mol­ecules into double chains, which are further linked by weak C—H⋯π inter­actions, forming a two-dimensional network.

## Related literature

For general background to Schiff base compounds in coord­ination chemistry, catalysis and enzymatic reactions, magnetism and mol­ecular architectures, see: Habibi *et al.* (2007 ▶). For the anti-bacterial properties of Schiff bases derived from 4-acyl-5-pyrazolones and their metal complexes, see: Li *et al.* (1997 ▶, 2004 ▶). For the anti-bacterial and biological activity of amino acid esters, see: Xiong *et al.* (1993 ▶). For related structures, see: Pettinari *et al.* (1994 ▶); Wang *et al.* (2003 ▶); Zhang *et al.* (2005 ▶); Zhu *et al.* (2005 ▶). For bond-length data, see: Allen *et al.* (1987 ▶).
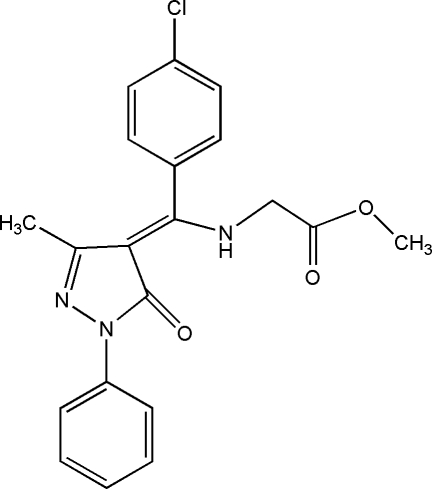


## Experimental

### 

#### Crystal data

C_20_H_18_ClN_3_O_3_
*M* *_r_* = 383.82
Triclinic, 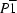

*a* = 9.309 (4) Å
*b* = 10.222 (4) Å
*c* = 10.685 (5) Å
α = 86.275 (8)°
β = 82.772 (8)°
γ = 71.749 (5)°
*V* = 957.6 (7) Å^3^
*Z* = 2
Mo *K*α radiation
μ = 0.23 mm^−1^
*T* = 296 K
0.24 × 0.20 × 0.18 mm

#### Data collection

Bruker APEXII CCD area-detector diffractometer
Absorption correction: multi-scan (*SADABS*; Sheldrick, 1996 ▶) *T*_min_ = 0.947, *T*_max_ = 0.960
4927 measured reflections
3364 independent reflections
1975 reflections with *I* > 2σ(*I*)
*R*_int_ = 0.019

#### Refinement

*R*[*F* ^2^ > 2σ(*F* ^2^)] = 0.075
*wR*(*F* ^2^) = 0.229
*S* = 1.05
3364 reflections
246 parameters
H-atom parameters constrained
Δρ_max_ = 0.54 e Å^−3^
Δρ_min_ = −0.44 e Å^−3^

### 

Data collection: *APEX2* (Bruker, 2003 ▶); cell refinement: *SAINT* (Bruker, 2001 ▶); data reduction: *SAINT*; program(s) used to solve structure: *SHELXS97* (Sheldrick, 2008 ▶); program(s) used to refine structure: *SHELXL97* (Sheldrick, 2008 ▶); molecular graphics: *SHELXTL* (Sheldrick, 2008 ▶) and *DIAMOND* (Brandenburg & Berndt, 1999 ▶); software used to prepare material for publication: *SHELXTL*.

## Supplementary Material

Crystal structure: contains datablocks I, global. DOI: 10.1107/S1600536809027858/hk2736sup1.cif

Structure factors: contains datablocks I. DOI: 10.1107/S1600536809027858/hk2736Isup2.hkl

Additional supplementary materials:  crystallographic information; 3D view; checkCIF report

## Figures and Tables

**Table 1:** Hydrogen-bond geometry (Å, °)

*D*—H⋯*A*	*D*—H	H⋯*A*	*D*⋯*A*	*D*—H⋯*A*
N3—H3⋯O1	0.86	2.06	2.755 (4)	138
N3—H3⋯O2	0.86	2.29	2.679 (4)	108
C16—H16⋯O1^i^	0.93	2.42	3.287 (5)	155
C17—H17⋯O1^ii^	0.93	2.54	3.359 (4)	147
C20—H20*B*⋯*Cg*3^iii^	0.96	2.69	3.604 (4)	160

Symmetry codes: (i) 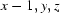; (ii) 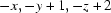; (iii) 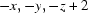. *Cg*3 is the centroid of the C12–C17 ring.

**Fig. 1.**
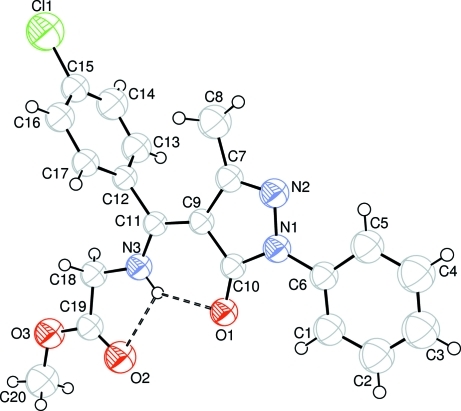
The molecular structure of the title molecule with the atom-numbering scheme. Displacement ellipsoids are drawn at the 30% probability level. Hydrogen bonds are shown as dashed lines.

**Fig. 2.**
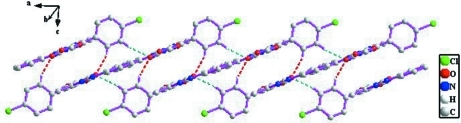
The one-dimensional plane formed by the intermolecular C–H···O hydrogen bonds.

**Fig. 3.**
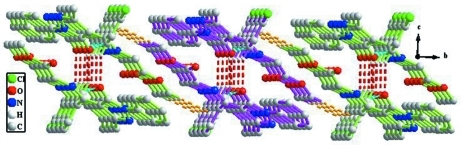
The two-dimensional network produced by the intermolecular C–H···π interactions.

## supplementary crystallographic information

### Comment

Schiff base compounds play an important role in the development of coordination chemistry related to catalysis and enzymatic reactions, magnetism, and molecular architectures [Habibi *et al.*, 2007]. In recent years, the Schiff bases derived from 4-acyl-5-pyrazolones and their metal complexes have been studied widely for their high antibacterial activation [Li *et al.*, 1997, 2004]. Amino acid esters also possess good antibacterial and biological activations [Xiong *et al.*, 1993]. Structures of Schiff bases derived from 4-acyl-5-pyrazolones and amino acid esters and closely related to the title compound have been reported [Zhu *et al., *2005; Zhang *et al., *2005]. We report herein the crystal structure of the title compound, (I).

In the molecule of the title compound, (I), (Fig. 1) the bond lengths (Allen *et al.*,  1987) and angles are within normal ranges. Rings A (C1-C6), B (N1/N2/C7/C9/C10) and C (C12-C17) are, of course, planar, and they are oriented at a dihedral angles of A/B = 4.13 (3), A/C = 81.79 (3) and B/C = 85.60 (3) °. Intramolecular N-H···O hydrogen bonds (Table 1) stabilize the enamine-keto form as in 4-{[3,4-dihydro-5-methyl-3-oxo-2-phenyl-2*H*-pyrazol-4-ylidene]-(phenyl)methyl]amino}-1,5-dimethyl-2-phenyl-1*H*-pyrazol-3(2*H*)-one, (II) (Wang *et al.*, 2003), and result in the formations of planar five- and six-membered rings: D (O2/N3/C18/C19/H3) and E (O1/N3/C9-C11/H3), in which the dihedral angle between them is D/E = 3.83 (4)°. Ring D is oriented with respect to the adjacent ring B at a dihedral angle of 3.12 (4)°. The dihedral angle between ring B and planar (O1/N3/C9-C11) moiety is 0.94 (3)°, which is reported as 3.56 (3)° in (II).

In the crystal structure, intermolecular C-H···O hydrogen bonds (Table 1) link the molecules into double chains (Fig. 2), in which they are further linked by weak C—H···π interactions (Table 1) to form a two-dimensional network (Fig. 3), in which they may be effective in the stabilization of the structure.

### Experimental

The title compound was synthesized by refluxing a mixture of 1-phenyl-3-methyl-4-(*p*-chlor-benzyl)-5-pyrazolone (15 mmol) (Pettinari *et al.*, 1994) and glycine methyl ester (15 mmol) in ethanol (100 ml) over a steam bath for about 5 h. The product was recrystallized from ethanol, affording pale yellow crystals suitable for X-ray analysis. Analysis calculated for C_20_H_18_ClN_3_O_3:_C 62.58, H 4.73, N 10.95%; found: C 62.55, H 4.70, N 10.91%.

### Refinement

H atoms were positioned geometrically with N-H = 0.86 Å (for NH) and C-H = 0.93, 0.97 and 0.96 Å, for aromatic, methylene and methyl H atoms, respectively, and constrained to ride on their parent atoms, with U_iso_(H) = xU_eq_(C,N), where x = 1.5 for methyl H and x = 1.2 for all other H atoms.

### Crystal data

**Table:**

C_20_H_18_ClN_3_O_3_	*Z* = 2
*M**_r_* = 383.82	*F*(000) = 400
Triclinic, *P*1	*D*_x_ = 1.331 Mg m^−^^3^
Hall symbol: -P 1	Mo *K*α radiation, λ = 0.71073 Å
*a* = 9.309 (4) Å	Cell parameters from 1323 reflections
*b* = 10.222 (4) Å	θ = 2.3–25.9°
*c* = 10.685 (5) Å	µ = 0.23 mm^−^^1^
α = 86.275 (8)°	*T* = 296 K
β = 82.772 (8)°	Block, colorless
γ = 71.749 (5)°	0.24 × 0.20 × 0.18 mm
*V* = 957.6 (7) Å^3^	

### Data collection

**Table:**

Bruker APEXII CCD area-detector diffractometer	3364 independent reflections
Radiation source: fine-focus sealed tube	1975 reflections with *I* > 2σ(*I*)
graphite	*R*_int_ = 0.019
φ and ω scans	θ_max_ = 25.0°, θ_min_ = 1.9°
Absorption correction: multi-scan (*SADABS*; Sheldrick, 1996)	*h* = −11→10
*T*_min_ = 0.947, *T*_max_ = 0.960	*k* = −11→12
4927 measured reflections	*l* = −11→12

### Refinement

**Table:**

Refinement on *F*^2^	Primary atom site location: structure-invariant direct methods
Least-squares matrix: full	Secondary atom site location: difference Fourier map
*R*[*F*^2^ > 2σ(*F*^2^)] = 0.075	Hydrogen site location: inferred from neighbouring sites
*wR*(*F*^2^) = 0.229	H-atom parameters constrained
*S* = 1.05	*w* = 1/[σ^2^(*F*_o_^2^) + (0.1339*P*)^2^] where *P* = (*F*_o_^2^ + 2*F*_c_^2^)/3
3364 reflections	(Δ/σ)_max_ < 0.001
246 parameters	Δρ_max_ = 0.54 e Å^−^^3^
0 restraints	Δρ_min_ = −0.44 e Å^−^^3^

### Special details

**Table:**

Geometry. All e.s.d.'s (except the e.s.d. in the dihedral angle between two l.s. planes) are estimated using the full covariance matrix. The cell e.s.d.'s are taken into account individually in the estimation of e.s.d.'s in distances, angles and torsion angles; correlations between e.s.d.'s in cell parameters are only used when they are defined by crystal symmetry. An approximate (isotropic) treatment of cell e.s.d.'s is used for estimating e.s.d.'s involving l.s. planes.
Refinement. Refinement of *F*^2^ against ALL reflections. The weighted *R*-factor *wR* and goodness of fit *S* are based on *F*^2^, conventional *R*-factors *R* are based on *F*, with *F* set to zero for negative *F*^2^. The threshold expression of *F*^2^ > σ(*F*^2^) is used only for calculating *R*-factors(gt) *etc*. and is not relevant to the choice of reflections for refinement. *R*-factors based on *F*^2^ are statistically about twice as large as those based on *F*, and *R*- factors based on ALL data will be even larger.

### Fractional atomic coordinates and isotropic or equivalent isotropic displacement parameters (Å^2^)

**Table:**

	*x*	*y*	*z*	*U*_iso_*/*U*_eq_	
Cl1	−0.55778 (16)	0.32750 (16)	0.61403 (15)	0.1056 (6)	
O1	0.2121 (3)	0.5392 (3)	0.8771 (2)	0.0536 (7)	
O2	0.2655 (4)	0.1981 (3)	1.0279 (3)	0.0771 (9)	
O3	0.1496 (3)	0.0367 (3)	1.0650 (3)	0.0716 (9)	
N1	0.1065 (3)	0.7252 (3)	0.7427 (3)	0.0520 (8)	
N2	−0.0161 (4)	0.7657 (3)	0.6679 (3)	0.0573 (9)	
N3	0.0591 (3)	0.3478 (3)	0.8789 (3)	0.0510 (8)	
H3	0.1341	0.3706	0.8998	0.061*	
C1	0.3191 (5)	0.7807 (4)	0.8132 (4)	0.0666 (12)	
H1	0.3399	0.7016	0.8647	0.080*	
C2	0.4086 (5)	0.8654 (5)	0.8087 (5)	0.0801 (14)	
H2	0.4895	0.8424	0.8574	0.096*	
C3	0.3824 (6)	0.9811 (6)	0.7354 (6)	0.0890 (15)	
H3A	0.4449	1.0368	0.7327	0.107*	
C4	0.2603 (7)	1.0159 (5)	0.6639 (5)	0.0880 (15)	
H4	0.2405	1.0960	0.6138	0.106*	
C5	0.1672 (5)	0.9319 (5)	0.6663 (4)	0.0697 (12)	
H5	0.0855	0.9552	0.6184	0.084*	
C6	0.1992 (4)	0.8118 (4)	0.7424 (3)	0.0525 (9)	
C7	−0.0797 (4)	0.6684 (4)	0.6807 (3)	0.0526 (10)	
C8	−0.2151 (5)	0.6816 (5)	0.6135 (4)	0.0776 (14)	
H8A	−0.2512	0.7733	0.5784	0.116*	
H8B	−0.2942	0.6634	0.6720	0.116*	
H8C	−0.1870	0.6165	0.5470	0.116*	
C9	−0.0021 (4)	0.5568 (4)	0.7624 (3)	0.0458 (9)	
C10	0.1185 (4)	0.5995 (4)	0.8021 (3)	0.0450 (8)	
C11	−0.0299 (4)	0.4347 (4)	0.8029 (3)	0.0432 (8)	
C12	−0.1577 (4)	0.3973 (4)	0.7612 (3)	0.0454 (9)	
C13	−0.1405 (5)	0.3365 (5)	0.6462 (4)	0.0683 (12)	
H13	−0.0457	0.3108	0.5986	0.082*	
C14	−0.2631 (6)	0.3136 (5)	0.6015 (4)	0.0770 (14)	
H14	−0.2513	0.2720	0.5244	0.092*	
C15	−0.4031 (5)	0.3528 (4)	0.6722 (4)	0.0591 (11)	
C16	−0.4215 (4)	0.4092 (4)	0.7886 (4)	0.0604 (11)	
H16	−0.5155	0.4314	0.8373	0.072*	
C17	−0.2987 (4)	0.4323 (4)	0.8322 (3)	0.0526 (10)	
H17	−0.3107	0.4719	0.9103	0.063*	
C18	0.0428 (4)	0.2179 (4)	0.9305 (4)	0.0545 (10)	
H18A	0.0493	0.1573	0.8625	0.065*	
H18B	−0.0560	0.2336	0.9794	0.065*	
C19	0.1662 (4)	0.1521 (4)	1.0130 (4)	0.0561 (10)	
C20	0.2628 (6)	−0.0381 (5)	1.1457 (5)	0.0911 (16)	
H20A	0.3615	−0.0631	1.0979	0.137*	
H20B	0.2404	−0.1199	1.1791	0.137*	
H20C	0.2620	0.0188	1.2140	0.137*	

### Atomic displacement parameters (Å^2^)

**Table:**

	*U*^11^	*U*^22^	*U*^33^	*U*^12^	*U*^13^	*U*^23^
Cl1	0.0921 (10)	0.1261 (12)	0.1282 (12)	−0.0573 (9)	−0.0643 (9)	0.0046 (9)
O1	0.0457 (14)	0.0636 (16)	0.0557 (15)	−0.0186 (12)	−0.0205 (12)	0.0069 (13)
O2	0.0713 (19)	0.075 (2)	0.098 (2)	−0.0316 (17)	−0.0458 (17)	0.0202 (17)
O3	0.0718 (19)	0.0580 (18)	0.091 (2)	−0.0220 (15)	−0.0336 (16)	0.0132 (16)
N1	0.0506 (18)	0.058 (2)	0.0516 (18)	−0.0206 (15)	−0.0159 (14)	0.0053 (15)
N2	0.0513 (18)	0.068 (2)	0.0531 (19)	−0.0161 (17)	−0.0172 (15)	0.0106 (16)
N3	0.0406 (16)	0.059 (2)	0.0574 (18)	−0.0180 (14)	−0.0197 (14)	0.0065 (16)
C1	0.060 (3)	0.066 (3)	0.080 (3)	−0.025 (2)	−0.021 (2)	0.006 (2)
C2	0.066 (3)	0.080 (3)	0.104 (4)	−0.032 (3)	−0.022 (3)	−0.001 (3)
C3	0.091 (4)	0.077 (3)	0.110 (4)	−0.044 (3)	−0.012 (3)	0.007 (3)
C4	0.107 (4)	0.074 (3)	0.089 (4)	−0.040 (3)	−0.016 (3)	0.024 (3)
C5	0.078 (3)	0.067 (3)	0.064 (3)	−0.022 (2)	−0.018 (2)	0.010 (2)
C6	0.054 (2)	0.055 (2)	0.049 (2)	−0.0177 (19)	−0.0023 (17)	−0.0072 (18)
C7	0.046 (2)	0.068 (3)	0.045 (2)	−0.018 (2)	−0.0144 (17)	0.0054 (19)
C8	0.067 (3)	0.101 (4)	0.074 (3)	−0.033 (3)	−0.038 (2)	0.031 (3)
C9	0.0382 (19)	0.061 (2)	0.0395 (18)	−0.0157 (17)	−0.0096 (15)	0.0030 (17)
C10	0.0407 (19)	0.052 (2)	0.0415 (19)	−0.0110 (16)	−0.0096 (15)	−0.0003 (17)
C11	0.0347 (17)	0.057 (2)	0.0381 (18)	−0.0138 (16)	−0.0038 (14)	−0.0054 (16)
C12	0.0424 (19)	0.055 (2)	0.0407 (18)	−0.0156 (17)	−0.0115 (15)	−0.0021 (16)
C13	0.058 (3)	0.098 (4)	0.052 (2)	−0.027 (2)	−0.0022 (19)	−0.022 (2)
C14	0.086 (3)	0.101 (4)	0.057 (3)	−0.038 (3)	−0.020 (2)	−0.020 (2)
C15	0.060 (3)	0.064 (3)	0.063 (3)	−0.026 (2)	−0.032 (2)	0.008 (2)
C16	0.042 (2)	0.071 (3)	0.069 (3)	−0.0180 (19)	−0.0127 (18)	−0.001 (2)
C17	0.040 (2)	0.068 (2)	0.049 (2)	−0.0129 (18)	−0.0076 (16)	−0.0108 (19)
C18	0.054 (2)	0.056 (2)	0.060 (2)	−0.0224 (19)	−0.0170 (19)	0.0012 (19)
C19	0.054 (2)	0.053 (2)	0.064 (3)	−0.017 (2)	−0.0137 (19)	−0.004 (2)
C20	0.093 (4)	0.068 (3)	0.111 (4)	−0.013 (3)	−0.053 (3)	0.027 (3)

### Geometric parameters (Å, °)

**Table:**

Cl1—C15	1.734 (4)	C7—C8	1.494 (5)
O1—C10	1.248 (4)	C8—H8A	0.9600
O2—C19	1.191 (4)	C8—H8B	0.9600
O3—C19	1.316 (5)	C8—H8C	0.9600
O3—C20	1.442 (5)	C9—C11	1.384 (5)
N1—C10	1.374 (5)	C9—C10	1.443 (5)
N1—C6	1.416 (5)	C11—C12	1.484 (5)
N1—N2	1.416 (4)	C12—C13	1.382 (5)
N2—C7	1.300 (5)	C12—C17	1.385 (5)
N3—C11	1.323 (4)	C13—C14	1.380 (6)
N3—C18	1.449 (5)	C13—H13	0.9300
N3—H3	0.8600	C14—C15	1.376 (6)
C1—C6	1.371 (5)	C14—H14	0.9300
C1—C2	1.372 (6)	C15—C16	1.373 (6)
C1—H1	0.9300	C16—C17	1.377 (5)
C2—C3	1.349 (7)	C16—H16	0.9300
C2—H2	0.9300	C17—H17	0.9300
C3—C4	1.389 (7)	C18—C19	1.498 (5)
C3—H3A	0.9300	C18—H18A	0.9700
C4—C5	1.396 (7)	C18—H18B	0.9700
C4—H4	0.9300	C20—H20A	0.9600
C5—C6	1.399 (6)	C20—H20B	0.9600
C5—H5	0.9300	C20—H20C	0.9600
C7—C9	1.449 (5)		
			
C19—O3—C20	115.9 (3)	O1—C10—C9	128.3 (3)
C10—N1—C6	130.1 (3)	N1—C10—C9	105.4 (3)
C10—N1—N2	111.5 (3)	N3—C11—C9	120.2 (3)
C6—N1—N2	118.3 (3)	N3—C11—C12	118.5 (3)
C7—N2—N1	106.7 (3)	C9—C11—C12	121.2 (3)
C11—N3—C18	126.5 (3)	C13—C12—C17	119.2 (3)
C11—N3—H3	116.8	C13—C12—C11	120.2 (3)
C18—N3—H3	116.8	C17—C12—C11	120.5 (3)
C6—C1—C2	120.5 (4)	C14—C13—C12	120.4 (4)
C6—C1—H1	119.7	C14—C13—H13	119.8
C2—C1—H1	119.7	C12—C13—H13	119.8
C3—C2—C1	121.7 (5)	C15—C14—C13	119.4 (4)
C3—C2—H2	119.2	C15—C14—H14	120.3
C1—C2—H2	119.2	C13—C14—H14	120.3
C2—C3—C4	118.9 (5)	C16—C15—C14	121.1 (4)
C2—C3—H3A	120.5	C16—C15—Cl1	119.6 (3)
C4—C3—H3A	120.5	C14—C15—Cl1	119.2 (3)
C3—C4—C5	120.8 (5)	C15—C16—C17	119.1 (4)
C3—C4—H4	119.6	C15—C16—H16	120.5
C5—C4—H4	119.6	C17—C16—H16	120.5
C4—C5—C6	118.6 (4)	C16—C17—C12	120.8 (3)
C4—C5—H5	120.7	C16—C17—H17	119.6
C6—C5—H5	120.7	C12—C17—H17	119.6
C1—C6—C5	119.5 (4)	N3—C18—C19	109.4 (3)
C1—C6—N1	122.0 (4)	N3—C18—H18A	109.8
C5—C6—N1	118.5 (4)	C19—C18—H18A	109.8
N2—C7—C9	111.6 (3)	N3—C18—H18B	109.8
N2—C7—C8	119.7 (3)	C19—C18—H18B	109.8
C9—C7—C8	128.7 (4)	H18A—C18—H18B	108.2
C7—C8—H8A	109.5	O2—C19—O3	125.3 (4)
C7—C8—H8B	109.5	O2—C19—C18	124.3 (4)
H8A—C8—H8B	109.5	O3—C19—C18	110.3 (3)
C7—C8—H8C	109.5	O3—C20—H20A	109.5
H8A—C8—H8C	109.5	O3—C20—H20B	109.5
H8B—C8—H8C	109.5	H20A—C20—H20B	109.5
C11—C9—C10	123.5 (3)	O3—C20—H20C	109.5
C11—C9—C7	131.6 (3)	H20A—C20—H20C	109.5
C10—C9—C7	104.9 (3)	H20B—C20—H20C	109.5
O1—C10—N1	126.3 (3)		
			
C10—N1—N2—C7	0.4 (4)	C7—C9—C10—N1	−1.0 (4)
C6—N1—N2—C7	177.9 (3)	C18—N3—C11—C9	178.9 (3)
C6—C1—C2—C3	0.0 (7)	C18—N3—C11—C12	−2.0 (5)
C1—C2—C3—C4	0.7 (8)	C10—C9—C11—N3	−2.2 (5)
C2—C3—C4—C5	−0.8 (8)	C7—C9—C11—N3	180.0 (4)
C3—C4—C5—C6	0.0 (7)	C10—C9—C11—C12	178.7 (3)
C2—C1—C6—C5	−0.8 (6)	C7—C9—C11—C12	0.8 (6)
C2—C1—C6—N1	178.6 (4)	N3—C11—C12—C13	−96.3 (4)
C4—C5—C6—C1	0.8 (6)	C9—C11—C12—C13	82.8 (5)
C4—C5—C6—N1	−178.7 (4)	N3—C11—C12—C17	88.6 (4)
C10—N1—C6—C1	−3.6 (6)	C9—C11—C12—C17	−92.2 (4)
N2—N1—C6—C1	179.3 (3)	C17—C12—C13—C14	1.4 (7)
C10—N1—C6—C5	175.8 (4)	C11—C12—C13—C14	−173.8 (4)
N2—N1—C6—C5	−1.2 (5)	C12—C13—C14—C15	0.5 (7)
N1—N2—C7—C9	−1.0 (4)	C13—C14—C15—C16	−2.7 (7)
N1—N2—C7—C8	179.4 (3)	C13—C14—C15—Cl1	178.5 (4)
N2—C7—C9—C11	179.4 (4)	C14—C15—C16—C17	3.0 (6)
C8—C7—C9—C11	−1.0 (7)	Cl1—C15—C16—C17	−178.2 (3)
N2—C7—C9—C10	1.3 (4)	C15—C16—C17—C12	−1.1 (6)
C8—C7—C9—C10	−179.2 (4)	C13—C12—C17—C16	−1.0 (6)
C6—N1—C10—O1	5.2 (6)	C11—C12—C17—C16	174.1 (4)
N2—N1—C10—O1	−177.6 (3)	C11—N3—C18—C19	−179.5 (3)
C6—N1—C10—C9	−176.7 (3)	C20—O3—C19—O2	−0.2 (6)
N2—N1—C10—C9	0.5 (4)	C20—O3—C19—C18	179.2 (4)
C11—C9—C10—O1	−1.3 (6)	N3—C18—C19—O2	−3.2 (6)
C7—C9—C10—O1	177.0 (3)	N3—C18—C19—O3	177.4 (3)
C11—C9—C10—N1	−179.3 (3)		

### Hydrogen-bond geometry (Å, °)

**Table:**

*D*—H···*A*	*D*—H	H···*A*	*D*···*A*	*D*—H···*A*
N3—H3···O1	0.86	2.06	2.755 (4)	138
N3—H3···O2	0.86	2.29	2.679 (4)	108
C16—H16···O1^i^	0.93	2.42	3.287 (5)	155
C17—H17···O1^ii^	0.93	2.54	3.359 (4)	147
C20—H20B···Cg3^iii^	0.96	2.69	3.604 (4)	160

Symmetry codes: (i) *x*−1, *y*, *z*; (ii) −*x*, −*y*+1, −*z*+2; (iii) −*x*, −*y*, −*z*+2.
